# Pre-Transplant Heavy Smoking Is Associated with Reduced Survival After Heart Transplantation Due to Infection and Malignancy

**DOI:** 10.3390/jcm14176024

**Published:** 2025-08-26

**Authors:** Karsten M. Heil, Rasmus Rivinius, Matthias Helmschrott, Ann-Kathrin Rahm, Philipp Ehlermann, Norbert Frey, Fabrice F. Darche

**Affiliations:** Department of Cardiology, Angiology and Pneumology, Heidelberg University Hospital, 69120 Heidelberg, Germany

**Keywords:** heart transplantation, pulmonary infection, mortality, lung cancer, pack-year, smoking

## Abstract

**Background**: Tobacco smoking is a known risk factor for adverse cardiovascular events. Many patients after heart transplantation (HTX) have a history of smoking, but the prognostic role of pre-transplant smoking remains uncertain. We thus investigated the effects of pre-transplant heavy smoking (≥20 pack-years) on outcomes after HTX. **Methods**: This observational retrospective single-centre study included 639 patients receiving HTX at Heidelberg Heart Center between 1989 and 2019. Patients were stratified by intensity of pre-transplant smoking (<20 pack-years or ≥20 pack-years). Analysis covered donor and recipient demographics, post-transplant medications, mortality including causes of death after HTX, and early post-transplant atrial fibrillation (AF) after HTX. **Results**: A total of 219 of the 639 HTX recipients (34.3%) had a pre-transplant history of heavy smoking (≥20 pack-years). These patients showed an increased 5-year post-transplant mortality (44.3% versus 28.6%, *p* < 0.001) and had a higher percentage of death due to infection/sepsis (21.5% versus 12.1%, *p* = 0.002) as well as due to malignancy (5.5% versus 1.7%, *p* = 0.007). Multivariate analysis demonstrated pre-transplant heavy smoking (≥20 pack-years) as an independent risk factor for five-year mortality after HTX (HR: 2.173, 95% CI: 1.601–2.950, *p* < 0.001). Analysis of secondary outcomes also showed a significantly higher rate of 30-day post-transplant AF (17.8% versus 11.7%, *p* = 0.032) in patients with a pre-transplant history of heavy smoking (≥20 pack-years). **Conclusions**: Pre-transplant heavy smoking is associated with early post-transplant AF, lung cancer, infection, and reduced survival after HTX.

## 1. Introduction

Tobacco smoking is a well-known modifiable risk factor for cardiovascular morbidity and mortality. It strongly contributes to the development of systemic inflammation, oxidative stress, endothelial dysfunction, and atherosclerosis, which in turn causes ischemic cardiomyopathy, peripheral artery disease, chronic obstructive pulmonary disease (COPD), and malignancies [1,2,3,4].

As the toxic effects of smoking grow cumulatively, the quantification of smoking is commonly expressed in pack-years, a composite metric reflecting both duration and intensity of smoking [5,6]. One pack-year is defined as smoking an average of 20 cigarettes (one pack) per day for one year [6]. Heavy smoking is commonly defined as ≥20 pack-years, an important threshold for clinical risk assessment [7,8,9,10].

Among patients with advanced heart failure who may require heart transplantation (HTX), a substantial subset has a history of smoking [11]. This topic remains highly relevant in the context of post-transplant care. An animal model using heterotopic rat HTX provided mechanistic insight into this matter by showing significantly reduced survival (*p* = 0.003) in HTX recipients with pre-transplant smoking exposure in comparison to HTX recipients without pre-transplant smoking exposure [12]. The authors concluded that potential mechanisms of pre-transplant smoking exposure comprise heightened systemic inflammation, alloimmune activation, and consequent myocardial as well as vascular destruction [12]. Therefore, in the light of limited organ supply and the detrimental effects of smoking, HTX guidelines consider active smoking as a contraindication for HTX candidacy and recommend a period of smoking cessation for a minimum of six months before being eligible for HTX listing [13,14,15].

Despite enforced pre-transplant smoking abstinence, differences remain in the duration and intensity of smoking exposure before HTX, as not all former smokers carry equal risk. HTX recipients with a pre-transplant history of light smoking (<20 pack-years) may demonstrate comparable post-transplant outcomes to HTX recipients who never smoked, while HTX recipients with a pre-transplant history of heavy smoking (≥20 pack-years) may exhibit disproportionately worse outcomes after HTX, even in the absence of post-transplant active smoking. Consequently, HTX recipients should not simply be analyzed by binary pre-transplant smoking status (smokers versus non-smokers before HTX) but rather by quantitative smoking exposure, for instance, non-smokers/light smokers (<20 pack-years) versus heavy smokers (≥20 pack-years) before HTX. This approach is even more important because data from the United Network for Organ Sharing (UNOS) registry lack granular pack year data [16]. We therefore sought to investigate the effects of pre-transplant heavy smoking (≥20 pack-years) on outcomes after HTX.

## 2. Patients and Methods

### 2.1. Patients

We carried out this study in compliance with the ethical principles set forth in the Declaration of Helsinki. The institutional review board (IRB) of Heidelberg University, Heidelberg, Germany, issued ethics approval number: S-286/2015, Version 1.2, 28 July 2020. Written informed consent was obtained from patients for enrollment in the Heidelberg HTX Registry and for the clinical and scientific use of their data. Because we only used routine clinical data—in accordance with the approved ethics protocol—no additional consent was required for this observational retrospective single-centre study [17,18,19,20].

We reviewed the medical data of all adult patients (≥18 years) who underwent HTX at Heidelberg Heart Center, Heidelberg, Germany, between 1989 and 2019, to determine pre-transplant tobacco smoking to ensure a minimum follow-up of five years after HTX. Individuals who had received a repeat HTX were excluded. Pre-transplant tobacco smoking was expressed in pack-years [5,6]. Patients were categorized into two cohorts: non-smokers or light smokers (<20 pack-years) before HTX, and heavy smokers (≥20 pack-years) before HTX. Following the definition of the US Preventive Services Task Force, those with ≥20 pack-years were classified as heavy smokers [7,8,9,10].

### 2.2. Follow-Up

Follow-up of HTX recipients adhered to the routine clinical protocol of Heidelberg Heart Center [17,18,19,20]. After discharge, patients visited the HTX outpatient clinic monthly during the first six post-transplant months, then every two months until the end of the first post-transplant year, and approximately three to four times per year thereafter, with extra appointments as clinically indicated [17,18,19,20].

Standard follow-up assessments consisted of medical history, physical examination, laboratory tests including immunosuppressive drug monitoring, resting 12-lead ECG, echocardiography, endomyocardial biopsy, an annual chest X-ray as well as an annual 24-h Holter recording. Complete follow-up data were available for all recipients; no patient was lost to follow-up [17,18,19,20].

### 2.3. Post-Transplant Pharmacotherapy

Post-transplant pharmacotherapy, including immunosuppressive regimens, followed the center’s standard [17,18,19,20]. Routine induction therapy after HTX consisted of an anti-thymocyte globulin-based immunosuppression. Cyclosporine A together with azathioprine served as the initial maintenance regimen early in the study period. From 2001 onward, azathioprine was replaced by mycophenolic acid, and starting in 2006, cyclosporine A was progressively replaced by tacrolimus. Patients also received steroids (prednisolone), which were gradually tapered and, when clinically feasible, discontinued six months after HTX [17,18,19,20].

### 2.4. Statistical Analysis

Data analysis was performed with MedCalc (Version 23.2.1, MedCalc Software Ltd., Ostend, Belgium). The results are presented as mean ± standard deviation (SD) or as count (*n*) with percentage (%).

Mean difference (MD) with 95% confidence interval (CI) was used for measures of association. Depending on data type and study question, statistical testing employed—as appropriate in each case—Student’s *t*-test, Mann–Whitney U-test, analysis of variance (ANOVA), Kruskal–Wallis test, chi-squared test, or Fisher’s exact test. Post-transplant survival between groups was graphically compared with Kaplan–Meier curves and the log-rank test. Figures were created with CorelDRAW Graphics Suite 2025 (Version 26.0.0.101; Corel Corporation, Ottawa, Ontario, Canada). A *p*-value of <0.050 denoted statistical significance [17,18,19,20].

Extensive univariate analyses were undertaken to detect differences between non-smokers/light smokers (<20 pack-years) and heavy smokers (≥20 pack-years) before HTX. Variables examined encompassed recipient data, recipient previous open-heart surgery, recipient principal diagnosis for HTX, donor data, transplant sex mismatch, perioperative data, immunosuppressive drug therapy, and post-transplant concomitant medications [17,18,19,20].

The primary outcome of this study was five-year mortality after HTX, comparing non-smokers/light smokers (<20 pack-years) before HTX with heavy smokers (≥20 pack-years) before HTX. Causes of death within five years after HTX were categorized into one of the following: graft failure, acute rejection, infection/sepsis, malignancy, or thromboembolic event/bleeding. Infection/sepsis was subdivided into pulmonary and abdominal infections. Malignancy was subdivided into lung cancer and other malignancies. A multivariate Cox regression model evaluated five-year mortality after HTX, incorporating fifteen variables which were statistically significant in univariate testing: recipient age, recipient male sex, recipient body mass index (BMI), recipient arterial hypertension, recipient dyslipidemia, recipient diabetes mellitus, recipient peripheral artery disease, recipient COPD, recipient chronic kidney disease (estimated glomerular filtration rate (eGFR) < 60 mL/min/1.73 m^2^), recipient overall open-heart surgery before HTX, recipient coronary artery bypass graft (CABG) surgery before HTX, recipient ischemic cardiomyopathy (CMP) as principal diagnosis for HTX, recipient non-ischemic CMP as principal diagnosis for HTX, recipient cardiac amyloidosis as principal diagnosis for HTX, and recipient heavy smoking (≥20 pack-years) before HTX. No further variables were added to the multivariate model to prevent biased coefficients and to preserve an adequate event to variable ratio [17,18,19,20].

Secondary outcomes comprised 30-day atrial fibrillation (AF) after HTX, 30-day rejection episode after HTX, 30-day TIA after HTX, 30-day stroke after HTX, five-year TIA after HTX, and five-year stroke after HTX, again comparing non-smokers/light smokers (<20 pack-years) with heavy smokers (≥20 pack-years) before HTX.

To evaluate potential era effects over the lengthy study period, a sensitivity analysis was performed in the subgroup treated with tacrolimus and mycophenolic acid, reflecting the immunosuppressive regimen change beginning in 2006 [17,18,19,20].

## 3. Results

### 3.1. Demographic and Clinical Characteristics

We included a total of 639 HTX recipients in this study after applying the exclusion criteria. A total of 219 of the 639 HTX recipients (34.3%) had a pre-transplant history of heavy smoking (≥20 pack-years), whereas 420 of the 639 HTX recipients (65.7%) had no pre-transplant history of heavy smoking (non-smokers or light smokers with <20 pack-years).

Patients with a pre-transplant history of heavy smoking (≥20 pack-years) had a higher recipient age (55.0 ± 7.2 years versus 50.5 ± 11.3 years, *p* < 0.001), a higher percentage of recipient male sex (86.3% versus 73.6%, *p* < 0.001), a higher recipient BMI (25.8 ± 4.1 kg/m^2^ versus 24.5 ± 3.9 kg/m^2^, *p* < 0.001), a higher percentage of arterial hypertension (65.8% versus 49.0%, *p* < 0.001), a higher percentage of dyslipidemia (78.5% versus 55.7%, *p* < 0.001), a higher percentage of diabetes mellitus (45.2% versus 27.6%, *p* < 0.001), a higher percentage of peripheral artery disease (37.0% versus 0.7%, *p* < 0.001), a higher percentage of COPD (60.3% versus 5.5%, *p* < 0.001), a higher percentage of chronic kidney disease (66.2% versus 53.1%, *p* = 0.001), a higher percentage of overall open-heart surgery before HTX (39.3% versus 24.8%, *p* < 0.001), a higher percentage of CABG surgery before HTX (20.1% versus 8.1%, *p* < 0.001), and a higher percentage of ischemic CMP as principal diagnosis for HTX (53.0% versus 22.2%, *p* < 0.001).

In contrast, patients with no pre-transplant history of heavy smoking (non-smokers or light smokers with <20 pack-years) had a higher percentage of non-ischemic CMP as principal diagnosis for HTX (60.2% versus 39.3%, *p* < 0.001), and a higher percentage of cardiac amyloidosis as principal diagnosis for HTX (12.1% versus 2.7%, *p* < 0.001). There were no statistically significant differences between both groups with respect to donor data, transplant sex mismatch, or perioperative data (all *p* ≥ 0.050). Demographic and clinical characteristics are given in Table 1.

### 3.2. Post-Transplant Medications

Comparison of the immunosuppressive drug therapy revealed no statistically significant differences between patients with a pre-transplant history of heavy smoking (≥20 pack-years) and patients with no pre-transplant history of heavy smoking (non-smokers or light smokers with <20 pack-years) regarding the use of cyclosporine A, tacrolimus, everolimus, azathioprine, mycophenolic acid, or steroids (all *p* ≥ 0.050). Likewise, we observed no statistically significant differences between both groups regarding the administration of acetylsalicylic acid, angiotensin-converting-enzyme inhibitors/angiotensin II receptor blockers, beta blockers, calcium channel blockers, diuretics, ivabradine, statins, or gastric protection drugs (all *p* ≥ 0.050). Post-transplant medications are presented in Table 2.

### 3.3. Post-Transplant Primary Outcome

In terms of the primary outcome of this study, patients with a pre-transplant history of heavy smoking (≥20 pack-years) showed a significantly higher one-year mortality after HTX (29.7% versus 19.5%, *p* = 0.004), two-year mortality after HTX (34.7% versus 23.6%, *p* = 0.003), and five-year mortality after HTX (44.3% versus 28.6%, *p* < 0.001). Details about the post-transplant primary outcome are provided in Table 3.

**Figure 1:**
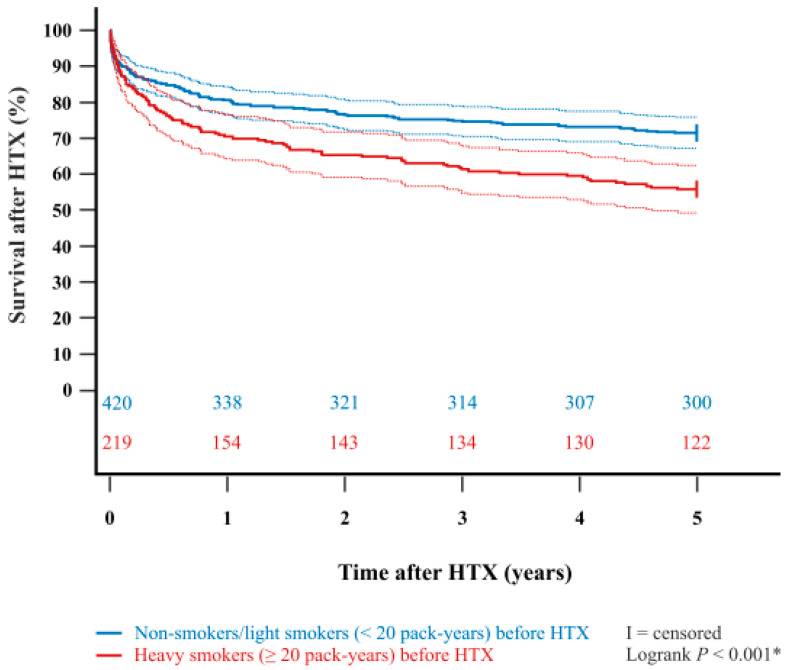
Five-year survival after HTX between non-smokers/light smokers (<20 pack-years) before HTX and heavy smokers (≥20 pack-years) before HTX (Kaplan–Meier estimator). Heavy smokers (≥20 pack-years) before HTX had a significantly lower five-year survival after HTX than non-smokers/light smokers (<20 pack-years) before HTX (*p* < 0.001). Dashed lines represent the 95% confidence interval around the respective survival curve. HTX = heart transplantation; * = statistically significant (*p* < 0.050).

**Figure 2:**
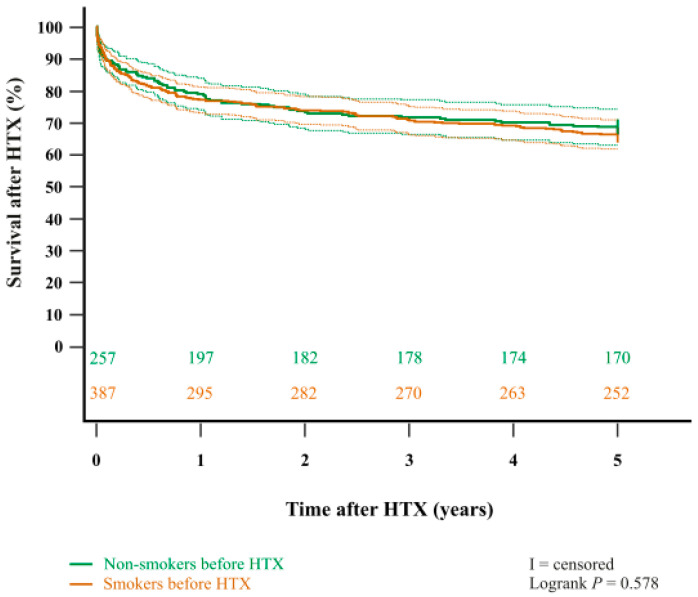
Five-year survival after HTX between non-smokers before HTX and smokers before HTX (Kaplan–Meier estimator). There was no statistically significant difference in five-year survival after HTX between non-smokers before HTX and smokers before HTX (*p* = 0.578). Dashed lines represent the 95% confidence interval around the respective survival curve. HTX = heart transplantation.

In addition, the Kaplan–Meier estimator showed a significantly worse five-year post-transplant survival (*p* < 0.001) in patients with a pre-transplant history of heavy smoking (≥20 pack-years) in comparison to patients with no pre-transplant history of heavy smoking (non-smokers or light smokers with <20 pack-years). Importantly, when stratified into patients who did not smoke before HTX at all (‘non-smokers before HTX’) and patients who used to smoke before HTX (‘smokers before HTX’), we found no statistically significant difference in five-year survival after (*p* = 0.578). Kaplan–Meier estimators are displayed in Figure 1 and Figure 2.

When examining causes of death, significantly more patients with a pre-transplant history of heavy smoking (≥20 pack-years) died from infection/sepsis (21.5% versus 12.1%, *p* = 0.002) and from malignancy (5.5% versus 1.7%, *p* = 0.002) within five years after HTX. Subgroup analysis of the categories infection/sepsis and malignancy showed that more patients with a pre-transplant history of heavy smoking (≥20 pack-years) died from pulmonary infection (14.2% versus 9.0%, *p* = 0.048), abdominal infection (7.3% versus 3.1%, *p* = 0.015), and from lung cancer (2.3% versus 0.3%, *p* = 0.011). No significant differences were observed between the two groups regarding graft failure, acute rejection, or thromboembolic event/bleeding at five-year follow-up after HTX (all *p* ≥ 0.050). Table 4 highlights the causes of death within five years after HTX.

Multivariate analysis for post-transplant mortality showed that pre-transplant history of heavy smoking (≥20 pack-years) was an independent risk factor for a more than twofold increased mortality within five years after HTX (HR: 2.173, 95% CI: 1.601–2.950, *p* < 0.001). In addition, recipient age (HR: 1.022, 95% CI: 1.006–1.039, *p* = 0.008), COPD (HR: 3.232, 95% CI: 2.210–4.727, *p* < 0.001), chronic kidney disease (HR: 1.414, 95% CI: 1.057–1.892, *p* = 0.020), and overall open-heart surgery before HTX (HR: 1.663, 95% CI: 1.135–2.436, *p* = 0.009) were independent risk factors for increased mortality within five years after HTX, whereas the other ten included variables (recipient BMI, recipient male sex, recipient arterial hypertension, recipient dyslipidemia, recipient diabetes mellitus, recipient peripheral artery disease, recipient CABG surgery before HTX, recipient ischemic CMP as principal diagnosis for HTX, recipient non-ischemic CMP as principal diagnosis for HTX, and recipient cardiac amyloidosis as principal diagnosis for HTX) had no statistically significant effect on five-year mortality after HTX. The multivariate analysis for five-year post-transplant mortality is shown in Table 5.

### 3.4. Post-Transplant Secondary Outcomes

In terms of secondary outcomes, patients with a pre-transplant history of heavy smoking (≥20 pack-years) had a significantly higher rate of 30-day AF after HTX (17.8% versus 11.7%, *p* = 0.032) than patients with no pre-transplant history of heavy smoking (non-smokers or light smokers with <20 pack-years). We observed no significant differences between both groups regarding 30-day TIA after HTX (0.0% versus 0.0%), 30-day stroke after HTX (2.3% versus 2.6%, *p* = 0.796), or 30-day rejection episode after HTX (19.2% versus 18.1%, *p* = 0.738).

### 3.5. Sensitivity Analysis

To account for the long study period, we performed a sensitivity analysis on a sub-group of patients to check for a possible era effect. This subgroup consisted of the 292 of the 639 (45.7%) HTX recipients who received tacrolimus and mycophenolic acid for immunosuppression. The analysis yielded comparable results, supporting the robustness of our findings and suggesting that an era effect was unlikely.

## 4. Discussion

### 4.1. The Role of Pre-Transplant Smoking as Risk Factor in HTX Recipients

Pre-transplant smoking has been associated with negative cardiovascular outcomes in HTX recipients including acute rejection, graft failure, infection, malignancy, and reduced post-transplant survival [16,21,22,23,24,25,26].

As the UNOS registry does not quantitatively capture cumulative smoking exposure in pack-years [16], we examined how pre-transplant heavy smoking (≥20 pack-years) influences post-transplant outcomes in a cohort of 639 HTX recipients. Pre-transplant heavy smoking (≥20 pack-years) was documented in 34.3% of our cohort. Compared with non-smokers or light smokers (<20 pack-years), these individuals were older, more often male, had a higher BMI, and exhibited higher rates of hypertension, dyslipidemia, diabetes mellitus, peripheral arterial disease, COPD, and chronic kidney disease; findings consistent with the well-established link between smoking and multiple cardiovascular risk factors [1,2,3,4]. Moreover, HTX recipients with pre-transplant heavy smoking (≥20 pack-years) had a markedly higher prevalence of prior CABG surgery (20.1% versus 8.1%), and more frequently suffered from ischemic CMP as principal diagnosis for HTX (53.0% versus 22.2%), supporting the role of smoking in the development of atherosclerosis [1,2,3,4].

Taken together, these findings suggest that the vasculopathic and cardiotoxic effects associated with pre-transplant smoking persist after HTX and may contribute to reduced post-transplant survival.

### 4.2. Pre-Transplant Smoking and Mortality

Given its detrimental effects, active smoking is considered a contraindication to HTX listing, and HTX programs generally mandate a minimum smoking abstinence period of six months before a patient is eligible for HTX listing [13,14,15]. This policy is supported by a study by Gali and colleagues [27] who performed a prospective observational multicenter study on waiting list mortality comparing the smoking status of 316 HTX candidates. They found a waiting list mortality of 42% in HTX candidates who reported active smoking at the time of HTX listing, compared with only 18% among those who had quit smoking and 14% among individuals who had never smoked [27]. The investigators also demonstrated a dose- and time-dependent relationship between smoking exposure and outcomes: former smokers who had abstained for over one year before HTX displayed mortality risks equivalent to never-smokers, highlighting the imperative for early pre-transplant smoking cessation to mitigate mortality [27].

In terms of post-transplant survival, a large study by Ohiomoba and colleagues [16] including 32,257 HTX recipients with complete data on history of smoking out of the 62,588 HTX recipients who are enrolled in the UNOS registry showed that HTX recipients with a pre-transplant history of smoking had a significantly higher one-year (11.6% versus 9.9%, *p* < 0.001) and five-year mortality after HTX (25.6% versus 20.9%, *p* < 0.001) than HTX recipients without a pre-transplant history of smoking. Dellgren and colleagues [24] described pre-transplant history of smoking as an independent predictor of long-term mortality (*p* = 0.034) in a cohort of 595 HTX recipients. Similarly, Sözen and colleagues [26] found that patients who were non-smokers before HTX survived longer than patients who were smokers before HTX in a cohort of 51 HTX recipients. Consistently, a recent comprehensive review by Annabi and colleagues [22] concluded that pre-transplant history of smoking is associated with inferior survival after HTX.

A slightly different approach was pursued by Sánchez-Lázaro and colleagues [25], who divided a cohort of 288 HTX recipients by their pre-transplant smoking status into three groups: non-smokers before HTX including those who quit smoking more than one year before HTX (*n* = 163), ex-smokers for less than one year before HTX (*n* = 76), and those who smoked until HTX (*n* = 49). They observed comparable survival rates between the non-smoker and the ex-smoker cohort (89.6% and 92.1%, respectively), whereas post-transplant survival in the smoker cohort was significantly diminished (81.6%, *p* = 0.031) [25].

A key limitation of these prior studies is that they did not quantify smoking exposure in pack-years or differentiate pre-transplant light smokers from heavy smokers but rather employed a binary smoker-versus-non-smoker classification before HTX [16,24,25]. Because HTX recipients with a pre-transplant history of light smoking (<20 pack-years) seem to exhibit post-transplant outcomes comparable to never-smokers, we concentrated our investigation on the influence of pre-transplant heavy smoking (≥20 pack-years) on outcomes after HTX. In our 639-patient cohort, HTX recipients with a pre-transplant history of heavy smoking (≥20 pack-years, *n* = 219) exhibited significantly elevated one-year (29.7% versus 19.5%, *p* = 0.004), two-year (34.7% versus 23.6%, *p* = 0.003), and five-year mortality after HTX (44.3% versus 28.6%, *p* < 0.001) relative to HTX recipients without pre-transplant history of heavy smoking (non-smokers or light smokers with <20 pack-years, *n* = 420). Additionally, pre-transplant history of heavy smoking (≥20 pack-years) independently conferred more than a two-fold increase in five-year mortality after HTX (HR: 2.173, 95% CI: 1.601–2.950, *p* < 0.001), even after multivariate adjustment for recipient age, recipient male sex, recipient BMI, and other cardiovascular risk factors.

Contrary to previous studies [16,24,25], our study detected no statistically significant difference in five-year survival after HTX between pre-transplant non-smokers and smokers (*p* = 0.578). This finding may stem from our comprehensive smoking-history assessments routinely performed for HTX candidates, in which even brief episodes of light smoking were recorded as positive smoking exposure, thereby yielding a heterogeneous pre-transplant smoker cohort with a wide spectrum of cumulative smoking burden.

Regarding the causes of death within 5 years after HTX, we found that significantly more patients with a pre-transplant history of heavy smoking (≥20 pack-years) died from infection/sepsis (21.5% versus 12.1%, *p* = 0.002) including pulmonary infection (14.2% versus 9.0%, *p* = 0.048). Smoking is the most common cause of COPD, a comorbidity linked to prolonged initial post-transplant hospitalization, delayed post-transplant extubation, as well as diminished post-transplant survival due to infection/sepsis and due to pulmonary infections in HTX recipients [19]. Similarly, Ohiomoba and colleagues [16] found that HTX recipients with a pre-transplant history of smoking experienced an increased risk of infection-related hospitalization (*p* < 0.001). In addition, Sánchez-Lázaro and colleagues [25] observed a prolonged intubation duration in the ex-smoker cohort, presumably attributable to smoking-related damage to pulmonary tissue and airways.

Our analysis also showed that significantly more HTX recipients with a pre-transplant history of heavy smoking (≥20 pack-years) died from malignancy (5.5% versus 1.7%, *p* = 0.002), especially from lung cancer (2.3% versus 0.3%, *p* = 0.011). This vulnerability to lung cancer in HTX recipients with a pre-transplant history of heavy smoking (≥20 pack-years) accords with the known tissue-damaging effects of smoking on the respiratory system, compounded by the lifelong need for immunosuppressive therapy after HTX [17,28,29].

Given the broad array of adverse effects—from pulmonary infections to lung cancer—it is unsurprising that a pre-transplant history of heavy smoking (≥20 pack-years) is linked to reduced survival after HTX, thereby underscoring the need for a stringent surveillance of these patients.

### 4.3. Cardiovascular Events After Heart Transplantation

Acute rejection and graft failure pose an imminent threat to HTX recipients with pre-transplant smoking as a suggested risk factor [12,16]. A large UNOS registry analysis by Ohiomoba and colleagues [16] found that any pre-transplant history of smoking heightened the odds of graft failure (odds ratio:1.23, *p* < 0.001) and acute rejection (odds ratio: 1.20, *p* < 0.001) after HTX.

In contrast, in our analysis of post-transplant cardiovascular events, we found no statistically significant differences between patients with a pre-transplant history of heavy smoking (≥20 pack-years) and patients without a pre-transplant history of heavy smoking (non-smokers or light smokers with <20 pack-years) in five-year mortality after HTX due to graft failure or acute rejection (all *p* ≥ 0.050). We also observed no statistically significant differences between both groups regarding the occurrence of a rejection episode within 30 days after HTX (*p* = 0.738). A plausible explanation for this discrepancy is the divergent quantification of smoking exposure: our cohort delineated heavy smokers (≥20 pack-years), whereas the UNOS registry analysis simply dichotomized patients by the presence or absence of any smoking history [16]. In terms of variations in how smoking history is classified, Sánchez-Lázaro and colleagues [25] found a reduced post-transplant survival in the smoker cohort (smoking until HTX) compared to the non-smoker cohort (including those abstinent for >1 year before HTX) and the ex-smoker cohort (<1 year of cessation before HTX) but observed no significant differences in the distribution of causes of death among the three groups.

A clinically important observation in our data set is the marked rise of 30-day AF after HTX (17.8% versus 11.7%, *p* = 0.032) in patients with a pre-transplant history of heavy smoking (≥20 pack-years). Smoking is a recognized risk factor for AF in the general population as demonstrated by the Atherosclerosis Risk in Communities (ARIC) cohort, which indicated that smoking was associated with a more than a two-fold increased risk of AF [30]. Moreover, patients with a pre-transplant history of heavy smoking (≥20 pack-years) in our cohort carried a greater burden of cardiovascular risk factors (such as higher age, diabetes mellitus, and COPD) that predispose to AF, potentially amplifying the observability of AF in this group [18,19,20].

In summary, our results extend the spectrum of smoking-related cardiovascular morbidity and mortality, including AF after HTX. Given the substantial increase in adverse outcomes after HTX, a cumulative exposure threshold (≥20 pack-years) should be integrated into pre-HTX-listing risk discussions. For HTX candidates with a history of heavy smoking, an extended period of smoking abstinence, routine cotinine testing, and low-dose chest computed tomography (CT) may be warranted. Former heavy smokers may also benefit from enhanced cancer screening protocols and proactive infection prophylaxis. Should former smokers resume smoking after HTX, this behavior should be promptly addressed by the transplant cardiologist to mitigate further cardiovascular and pulmonary complications after HTX. Additionally, lifelong smoking relapse-prevention programs may also be beneficial especially for those patients.

### 4.4. Study Limitations

The present study derives its findings from the Heidelberg HTX Registry, a large single-center database. Because this research design is intrinsically subject to certain constraints, the findings should be interpreted cautiously and placed within the broader corpus of published research. Nonetheless, our analysis used granular clinical information from 639 HTX recipients managed under standardized treatment and follow-up protocols, thereby reducing the likelihood of selection bias and confounding variables [17,18,19,20].

To secure an adequate sample size for dependable statistical inference, we enrolled patients who underwent HTX at the Heidelberg Heart Center between 1989 and 2019 and observed them for up to five years. Owing to the protracted study interval, temporal shifts in surgical techniques and medical management (i.e., era effects) may have influenced the results. To mitigate this concern, we performed a sensitivity analysis limited to recipients treated with tacrolimus and mycophenolic acid, the standard immunosuppressive regimen introduced at the Heidelberg Heart Center from 2006 onward. The robustness of the overall study results is supported by the consistent findings in this subgroup [17,18,19,20].

This study did not capture data on recipients’ smoking status after HTX, nor were objective measures (for instance, urine cotinine levels) available to verify continued abstinence. The lack of post-transplant smoking data limits our ability to assess the impact of any smoking relapse on outcomes after HTX and could lead to underestimation of the true effect of smoking on post-transplant events. This limitation underscores the need for future studies to incorporate post-transplant smoking surveillance when evaluating long-term outcomes in HTX recipients. Furthermore, it is important to regard our results as hypothesis-generating, especially regarding post-transplant survival, which can be impacted by a variety of factors. Although we detected an association between pre-transplant heavy smoking (≥20 pack-years) and increased post-transplant mortality, these findings cannot by themselves establish causality given the limitations of our study design. Large-scale, prospective multicenter studies are needed to confirm these observations and clarify the underlying mechanisms [17,18,19,20].

## 5. Conclusions

Tobacco smoking is a well-established risk marker for adverse cardiovascular events. Active smoking is a contraindication for HTX listing and HTX candidates need to be abstinent from smoking for at least six months prior to HTX listing. As many HTX recipients have a history of smoking, we investigated the effects of pre-transplant heavy smoking (≥20 pack-years) on outcomes after HTX. In this retrospective, single-center observational study, we analyzed a cohort of 639 patients who underwent HTX at the Heidelberg Heart Center between 1989 and 2019. Among these, 219 HTX recipients (34.3%) had a pre-transplant history of heavy smoking (≥20 pack-years). Patients with a pre-transplant history of heavy smoking (≥20 pack-years) showed a significantly higher five-year post-transplant mortality (44.3% versus 28.6%, *p* < 0.001). Additionally, this group showed increased rates of death due to infection/sepsis (21.5% versus 12.1%, *p* = 0.002) and malignancy (5.5% versus 1.7%, *p* = 0.002). Multivariate analysis identified pre-transplant heavy smoking (≥20 pack-years) as an independent risk factor for five-year mortality (HR: 2.173, 95% CI: 1.601–2.950, *p* < 0.001). Analysis of secondary outcomes revealed a significantly higher rate of 30-day post-transplant AF (17.8% versus 11.7%, *p* = 0.032) in patients with a pre-transplant history of heavy smoking (≥20 pack-years).

In summary, our findings indicate that pre-transplant heavy smoking (≥20 pack-years) is linked with early post-transplant AF, lung cancer, infection, and reduced survival after HTX. These results suggest that a pre-transplant history of heavy smoking (≥20 pack-years) may serve as a valuable and easily accessible marker for identifying HTX candidates at high risk. Preventive measures including the close monitoring and management of cardiovascular risk factors are warranted in these high-risk patients. If our findings are confirmed in larger studies, pre-transplant heavy smoking status (≥20 pack-years)—even if ceased for a minimum of six months before HTX—might warrant careful consideration in HTX candidate selection given the high mortality risk.

## Figures and Tables

**Table 1:** Demographic and clinical characteristics.

Parameter	All Patients (*n* = 639)	Non-Smokers/ Light Smokers (<20 Pack-Years) Before HTX (*n* = 420)	Heavy Smokers (≥20 Pack-Years) Before HTX (*n* = 219)	Difference	95% CI	*p*-Value	
Recipient data							
Age (years), mean ± SD	52.1 ± 10.3	50.5 ± 11.3	55.0 ± 7.2	4.5	3.0–6.0	<0.001	*
Male sex, *n* (%)	498 (77.9%)	309 (73.6%)	189 (86.3%)	12.7%	6.5–18.9%	<0.001	*
BMI (kg/m^2^), mean ± SD	24.9 ± 4.0	24.5 ± 3.9	25.8 ± 4.1	1.3	0.6–2.0	<0.001	*
Arterial hypertension, *n* (%)	350 (54.8%)	206 (49.0%)	144 (65.8%)	16.8%	8.9–24.7%	<0.001	*
Dyslipidemia, *n* (%)	406 (63.5%)	234 (55.7%)	172 (78.5%)	22.8%	15.6–30.0%	<0.001	*
Diabetes mellitus, *n* (%)	215 (33.6%)	116 (27.6%)	99 (45.2%)	17.6%	9.7–25.5%	<0.001	*
Peripheral artery disease, *n* (%)	84 (13.1%)	3 (0.7%)	81 (37.0%)	36.3%	29.9–42.7%	<0.001	*
COPD, *n* (%)	155 (24.3%)	23 (5.5%)	132 (60.3%)	54.8%	48.0–61.6%	<0.001	*
Chronic kidney disease ^, *n* (%)	368 (57.6%)	223 (53.1%)	145 (66.2%)	13.1%	5.2–21.0%	0.001	*
eGFR (ml/min/1.73 m^2^), mean ± SD	60.3 ± 21.7	61.3 ± 23.0	58.2 ± 18.8	3.1	−0.2–6.4	0.069	
Previous open-heart surgery							
Overall open-heart surgery, *n* (%)	190 (29.7%)	104 (24.8%)	86 (39.3%)	14.5%	6.8–22.2%	<0.001	*
CABG surgery, *n* (%)	78 (12.2%)	34 (8.1%)	44 (20.1%)	12.0%	6.1–17.9%	<0.001	*
Other surgery °, *n* (%)	71 (11.1%)	42 (10.0%)	29 (13.2%)	3.2%	−2.1–8.5%	0.216	
VAD surgery, *n* (%)	55 (8.6%)	33 (7.9%)	22 (10.0%)	2.1%	−2.6–6.8%	0.349	
Principal diagnosis for HTX							
Ischemic CMP, *n* (%)	209 (32.7%)	93 (22.2%)	116 (53.0%)	30.8%	23.1–38.5%	<0.001	*
Non-ischemic CMP, *n* (%)	339 (53.1%)	253 (60.2%)	86 (39.3%)	20.9%	12.9–28.9%	<0.001	*
Valvular heart disease, *n* (%)	34 (5.3%)	23 (5.5%)	11 (5.0%)	0.5%	−3.1–4.1%	0.809	
Cardiac amyloidosis, *n* (%)	57 (8.9%)	51 (12.1%)	6 (2.7%)	9.4%	5.6–13.2%	<0.001	*
Donor data							
Age (years), mean ± SD	41.0 ± 13.4	40.5 ± 13.1	42.0 ± 13.9	1.5	−0.7–3.7	0.163	
Male sex, *n* (%)	278 (43.5%)	176 (41.9%)	102 (46.6%)	4.7%	−3.4–12.8%	0.258	
BMI (kg/m^2^), mean ± SD	24.8 ± 4.1	24.7 ± 4.2	25.1 ± 3.8	0.4	−0.2–1.0	0.255	
Transplant sex mismatch							
Mismatch, *n* (%)	283 (44.3%)	183 (43.6%)	100 (45.6%)	2.0%	−6.1–10.1%	0.614	
Donor (m) to recipient (f), *n* (%)	31 (4.9%)	25 (6.0%)	6 (2.7%)	3.3%	−0.1–6.7%	0.073	
Donor (f) to recipient (m), *n* (%)	252 (39.4%)	158 (37.6%)	94 (42.9%)	5.3%	−2.7–13.3%	0.193	
Perioperative data							
Ischemic time (min), mean ± SD	223.4 ± 68.4	222.2 ± 66.3	225.7 ± 72.5	3.5	−8.0–15.0	0.558	
Biatrial anastomosis, *n* (%)	164 (25.7%)	101 (24.0%)	63 (28.7%)	4.7%	−2.6–12.0%	0.195	
Bicaval anastomosis, *n* (%)	198 (31.0%)	130 (31.0%)	68 (31.1%)	0.1%	−7.5–7.7%	0.980	
Total orthotopic anastomosis, *n* (%)	277 (43.3%)	189 (45.0%)	88 (40.2%)	4.8%	−3.3–12.9%	0.243	

BMI = body mass index; CABG = coronary artery bypass graft; CI = confidence interval; CMP = cardiomyopathy; COPD = chronic obstructive pulmonary disease; f = female; eGFR = estimated glomerular filtration rate; HTX = heart transplantation; m = male; *n* = number; SD = standard deviation; VAD = ventricular assist device; ^ = eGFR < 60 mL/min/1.73 m^2^; ° = congenital, valvular, or ventricular surgery; * = statistically significant (*p* < 0.050).

**Table 2:** Post-transplant medications.

Parameter	All Patients (*n* = 639)	Non-Smokers/ Light Smokers (<20 Pack-Years) Before HTX (*n* = 420)	Heavy Smokers (≥20 Pack-Years) Before HTX (*n* = 219)	Difference	95% CI	*p*-Value
Immunosuppressive drug therapy						
Cyclosporine A, *n* (%)	347 (54.3%)	229 (54.5%)	118 (53.9%)	0.6%	−7.5–8.7%	0.877
Tacrolimus, *n* (%)	292 (45.7%)	191 (45.5%)	101 (46.1%)	0.6%	−7.5–8.7%	0.877
Azathioprine, *n* (%)	267 (41.8%)	166 (39.5%)	101 (46.1%)	6.6%	−1.5–14.7%	0.109
Mycophenolic acid, *n* (%)	372 (58.2%)	254 (60.5%)	118 (53.9%)	6.6%	−1.5–14.7%	0.109
Steroids, *n* (%)	639 (100.0%)	420 (100.0%)	219 (100.0%)	0.0%	n. a.	n. a.
Concomitant medications						
ASA, *n* (%)	68 (10.6%)	38 (9.0%)	30 (13.7%)	4.7%	−0.6–10.0%	0.070
Beta blocker, *n* (%)	114 (17.8%)	73 (17.4%)	41 (18.7%)	1.3%	−5.0–7.6%	0.674
Ivabradine, *n* (%)	61 (9.5%)	43 (10.2%)	18 (8.2%)	2.0%	−2.7–6.7%	0.410
Calcium channel blocker, *n* (%)	171 (26.8%)	105 (25.0%)	66 (30.1%)	5.1%	−2.3–12.5%	0.164
ACE inhibitor/ARB, *n* (%)	278 (43.5%)	185 (44.0%)	93 (42.5%)	1.5%	−6.6–9.6%	0.702
Diuretic, *n* (%)	639 (100.0%)	420 (100.0%)	219 (100.0%)	0.0%	n. a.	n. a.
Statin, *n* (%)	254 (39.7%)	178 (42.4%)	76 (34.7%)	7.7%	−0.2–15.6%	0.060
Gastric protection drug ^†^, *n* (%)	639 (100.0%)	420 (100.0%)	219 (100.0%)	0.0%	n. a.	n. a.

ACE inhibitor = angiotensin-converting-enzyme inhibitor; ARB = angiotensin II receptor blocker; ASA = acetylsalicylic acid; CI = confidence interval; *n* = number; n. a. = not applicable; ^†^ = gastric protection drug defined as proton pump inhibitor (PPI) or histamine receptor (H_2_) blocker.

**Table 3:** Post-transplant primary outcome.

Parameter	All Patients (*n* = 639)	Non-Smokers/ Light Smokers (<20 Pack-Years) Before HTX (*n* = 420)	Heavy Smokers (≥20 Pack-Years) Before HTX (*n* = 219)	Difference	95% CI	*p*-Value	
30-day mortality after HTX, *n* (%)	64 (10.0%)	39 (9.3%)	25 (11.4%)	2.1%	−2.9–7.1%	0.395	
1-year mortality after HTX, *n* (%)	147 (23.0%)	82 (19.5%)	65 (29.7%)	10.2%	3.1–17.3%	0.004	*
2-year mortality after HTX, *n* (%)	175 (27.4%)	99 (23.6%)	76 (34.7%)	11.1%	3.6–18.6%	0.003	*
5-year mortality after HTX, *n* (%)	217 (34.0%)	120 (28.6%)	97 (44.3%)	15.7%	7.8–23.6%	<0.001	*

CI = confidence interval; HTX = heart transplantation; *n* = number; * = statistically significant (*p* < 0.050).

**Table 4:** Causes of death within five years after HTX.

Parameter	All Patients (*n* = 639)	Non-Smokers/Light Smokers (<20 Pack-Years) Before HTX (*n* = 420)	Heavy Smokers (≥20 Pack-Years) Before HTX (*n* = 219)	Difference	95% CI	*p*-Value	
Graft failure, *n* (%)	75 (11.7%)	44 (10.5%)	31 (14.2%)	3.7%	−1.8–9.2%	0.170	
Acute rejection, *n* (%)	8 (1.3%)	7 (1.7%)	1 (0.5%)	1.2%	−0.4–2.8%	0.192	
Infection/Sepsis, *n* (%)	98 (15.3%)	51 (12.1%)	47 (21.5%)	9.4%	3.1–15.7%	0.002	*
Pulmonary infection, *n* (%)	69 (10.8%)	38 (9.0%)	31 (14.2%)	5.2%	0.1–10.3%	0.048	*
Abdominal infection, *n* (%)	29 (4.5%)	13 (3.1%)	16 (7.3%)	4.2%	0.4–8.0%	0.015	*
Malignancy, *n* (%)	19 (3.0%)	7 (1.7%)	12 (5.5%)	3.8%	0.5–7.1%	0.007	*
Lung cancer, *n* (%)	6 (1.0%)	1 (0.3%)	5 (2.3%)	2.0%	0.1–3.9%	0.011	*
Other malignancies, *n* (%)	13 (2.0%)	6 (1.4%)	7 (3.2%)	1.8%	−0.8–4.4%	0.133	
Thromboembolic event/bleeding, *n* (%)	17 (2.7%)	11 (2.6%)	6 (2.7%)	0.1%	−2.5–2.7%	0.928	
All causes, *n* (%)	217 (34.0%)	120 (28.6%)	97 (44.3%)	15.7%	7.8–23.6%	<0.001	*

CI = confidence interval; HTX = heart transplantation; *n* = number; * = statistically significant (*p* < 0.050).

**Table 5:** Multivariate analysis for five-year mortality after HTX.

Parameter	Hazard Ratio	95% CI	*p*-Value	
Recipient age (years)	1.022	1.006–1.039	0.008	*
Recipient male sex (in total)	0.868	0.617–1.221	0.416	
Recipient BMI (kg/m^2^)	0.990	0.954–1.027	0.576	
Arterial hypertension (in total)	0.739	0.502–1.087	0.124	
Dyslipidemia (in total)	0.708	0.488–1.028	0.069	
Diabetes mellitus (in total)	1.095	0.806–1.486	0.562	
Peripheral artery disease (in total)	1.033	0.764–1.396	0.834	
COPD (in total)	3.232	2.210–4.727	<0.001	*
Chronic kidney disease ^ (in total)	1.414	1.057–1.892	0.020	*
Overall open-heart surgery ^†^ (in total)	1.663	1.135–2.436	0.009	*
CABG surgery ^†^ (in total)	1.062	0.718–1.571	0.763	
Ischemic CMP ° (in total)	1.255	0.644–2.447	0.505	
Non-ischemic CMP ° (in total)	0.688	0.386–1.226	0.205	
Cardiac amyloidosis ° (in total)	1.154	0.549–2.426	0.706	
Heavy smoking (≥20 pack-years) before HTX (in total)	2.173	1.601–2.950	<0.001	*

BMI = body mass index; CABG = coronary artery bypass graft; CI = confidence interval; CMP = cardiomyopathy; COPD = chronic obstructive pulmonary disease; HTX = heart transplantation; ^ = eGFR < 60 mL/min/1.73 m^2^; ^†^ = before HTX; ° = as principal diagnosis for HTX; * = statistically significant (*p* < 0.050).

## Data Availability

The original contributions presented in this study are included in the article, further inquiries can be directed to the corresponding author.
